# Natriuretic peptides and cGMP in metabolic disease

**DOI:** 10.1186/2050-6511-16-S1-A7

**Published:** 2015-09-02

**Authors:** Sheila Collins

**Affiliations:** 1 Sanford-Burnham Medical Research Institute, Orlando, FL 32827, USA

## 

The cardiac natriuretic peptides ANP and BNP stimulate adipocyte metabolism to increase lipolysis of triglycerides to free fatty acids, as well promote brown adipocyte energy expenditure through uncoupled mitochondrial respiration. Energy expenditure in brown adipocytes holds potential for improving cardiometabolic disease by consuming glucose and fatty acids and thereby improving insulin sensitivity. This presentation will describe studies in mouse models lacking the natriuretic peptide ‘clearance receptor’ Nprc (gene = Npr3) in adipose and other tissues, as well as more clinically oriented studies in human subjects examining a role for the natriuretic peptide system in insulin sensitivity and energy expenditure. We will also describe novel signal transduction mechanisms downstream of PKA and PKG that promote the increase in brown adipocytes within white adipose tissue and their metabolic activation.

